# EEG hyper-connectivity in high-risk infants is associated with later autism

**DOI:** 10.1186/1866-1955-6-40

**Published:** 2014-11-07

**Authors:** Elena V Orekhova, Mayada Elsabbagh, Emily JH Jones, Geraldine Dawson, Tony Charman, Mark H Johnson, The BASIS Team

**Affiliations:** Centre for Brain and Cognitive Development, School of Psychology, Birkbeck, University of London, Henry Welcome Building, London, WC1E 7HX UK; Department of Psychiatry, McGill University, Montreal, PQ H3A 1A1 Canada; Department of Psychiatry and Behavioral Sciences, School of Medicine, Duke University, Durham, NC 27705 USA; Department of Psychology, Institute of Psychiatry, Psychology & Neuroscience, King’s College London, London, SE5 8AF UK

**Keywords:** Autism spectrum disorders, EEG, Connectivity, Alpha, Infants, Siblings

## Abstract

**Background:**

It has been previously reported that structural and functional brain connectivity in individuals with autism spectrum disorders (ASD) is atypical and may vary with age. However, to date, no measures of functional connectivity measured within the first 2 years have specifically associated with a later ASD diagnosis.

**Methods:**

In the present study, we analyzed functional brain connectivity in 14-month-old infants at high and low familial risk for ASD using electroencephalography (EEG). EEG was recorded while infants attended to videos. Connectivity was assessed using debiased weighted phase lag index (dbWPLI). At 36 months, the high-risk infants were assessed for symptoms of ASD.

**Results:**

As a group, high-risk infants who were later diagnosed with ASD demonstrated elevated phase-lagged alpha-range connectivity as compared to both low-risk infants and high-risk infants who did not go on to ASD. Hyper-connectivity was most prominent over frontal and central areas. The degree of hyper-connectivity at 14 months strongly correlated with the severity of restricted and repetitive behaviors in participants with ASD at 3 years. These effects were not attributable to differences in behavior during the EEG session or to differences in spectral power.

**Conclusions:**

The results suggest that early hyper-connectivity in the alpha frequency range is an important feature of the ASD neurophysiological phenotype.

**Electronic supplementary material:**

The online version of this article (doi:10.1186/1866-1955-6-40) contains supplementary material, which is available to authorized users.

## Background

Autism spectrum disorder (ASD) is primarily characterized by impairments in social communication skills and the presence of repetitive and stereotypical behaviors [1]. Recent research shows that many genes implicated in ASD are involved in formation and regulation of synaptic pathways and neural connections [2, 3]. Given that the pruning and modification of such synapses and neural pathways are partly shaped by experience [4, 5], studies of brain connectivity in very young children with ASD, or in infants ‘at risk’ for developing ASD, are critical.

Structural and functional MRI studies in adults with ASD have predominantly reported weaker long-range cortico-cortical connections [6, 7]. The studies that used electro- (EEG) and magneto-encephalography (MEG) also primarily found reduced functional connectivity, although the results were more variable (see Additional file 1). In contrast, more recent fMRI studies in younger (pre-pubertal) children with ASD have reported *increased* functional connectivity in brain networks [8–10]. Further, functional hyper-connectivity associates with greater severity of autism symptoms in children [8]. In line with the increased functional connectivity findings, recent diffusion tensor imaging studies revealed that young children and toddlers with ASD have atypically early maturation of white matter [11–14].

The presence of different patterns of connectivity abnormalities in children and adults with ASD may reflect an atypical trajectory of brain development. The early overgrowth of white and gray matter during infancy and toddlerhood in individuals with ASD is followed by normal or decreased growth during later childhood [15–18]. This pattern of atypical development seems to be a particular feature of the temporal, frontal, and cingulate cortices that play crucial roles in attention, emotions, and social cognition [15, 18–21]. It is possible that the early increases in fMRI connectivity reported in younger children with ASD reflect their early brain overgrowth. On the other hand, the under-connectivity reported in adolescences and adults with ASD may be linked to the reduced brain growth during later childhood and/or to some compensatory processes.

Siblings of children with ASD have an increased risk of developing the disorder [22]. Therefore, studies in infant siblings of children with ASD allow one to study the early signs of the disorder before reliable diagnosis is obtained in later childhood. A recent longitudinal study found atypical developmental trajectories of cortical fiber tracts in at-risk infants who displayed ASD features at 2 years of life [14]. Specifically, development of most fiber tracts studied in the infants with later ASD was characterized by higher fractional anisotropy (FA) values at 6 months, followed by slower change over time relative to high-risk infants with few autistic traits assessed at 24 months. Thus, by 24 months of age, those with emerging ASD had lower FA values.

Structural and functional connectivity are not unequivocally associated [23]. It is therefore unclear if, and how, early differences in structural connectivity might affect emerging functional connections to result in later diagnosed ASD or, indeed, how early differences in functional connectivity might sculpt later appearing differences in structural connectivity. While in adults functional connectivity has been mainly investigated with fMRI, this method has practical limitations in infants and young children. Even in the youngest children, however, functional connectivity can be measured noninvasively with EEG. While motion inside the MRI scanner may create artifacts presenting a serious problem for analysis of connectivity [24], EEG connectivity is unlikely to be affected by motion in a similar adverse way because the EEG electrodes are in fixed locations on the scalp. In addition, the high time resolution of the method allows one to measure phase coupling of the oscillatory EEG signals indicating synchronization of neuronal populations on a millisecond timescale [25].

Although EEG has been widely used to investigate possible neural markers of ASD in adults and children, only two studies have investigated ‘ongoing’ EEG in infants at risk for later autism [26, 27], and neither of these measured connectivity. Tierney and colleagues [27] and Bosl and colleagues [26] describe differences in quantitative EEG features between infants at high and low familial risk for ASD (i.e., infants who had an older sibling with ASD), but the actual outcome of infants ‘at risk’ was not known at the time of investigation.

In the present study, we used high-density EEG to examine functional connectivity in a cohort of 14-month-old infants at low or high familial risk for ASD and then assessed symptoms of ASD at age 3 years. Different frequencies of EEG and MEG oscillations are associated with different cognitive, emotional, and motor processes [25, 28, 29] and may reflect activity in different brain networks. Further, atypical EEG connectivity in neuropsychiatric disorders may be specific to particular frequency bands [30, 31]. In this study, we recorded EEG while infants attended to a series of videos and then analyzed connectivity within the infant alpha band. There were several reasons for focusing on the alpha frequency band. First, the alpha rhythm is intimately related to attention processes in adults [28, 32] and infants [33] and alpha-range connectivity increases during states of attentiveness [32, 34]. Therefore, we expected the presence of reliable alpha-range connectivity during sustained attention to the videos in our study. Second, alpha activity is to a lesser extent than theta activity modulated by inter-individual differences in emotional and cognitive engagement [35, 36]. Therefore, we expected that the inter-individual variations in these uncontrolled factors during passive viewing of the videos would contribute less to the alpha then into the theta frequency band. Third, the presence of alpha peak in the infant EEG power spectra suggests the presence of distinct rhythmic processes at this frequency and, correspondingly, good signal-to-noise ratio. Fourth, alpha activity is less prone to contamination by muscle artifacts than the EEG signal of higher frequencies [37, 38] and is less prone to contamination by movement artifacts than the delta activity. Considering all these factors, we hypothesized that in infants any altered coupling between cortical regions during attention to videos would be most reliably detected in this frequency band.

The magnitude of alpha-range connectivity can be modulated by a participant’s functional state and behavior [34, 39, 40]. In order to assess the effect of ongoing behavioral state on EEG connectivity, and to control for possible outcome group differences in behavior, we complemented the EEG analysis with an analysis of infants’ behavior during the EEG recording session.

Siegel et al. [25] point to several important challenges for M/EEG studies of functional brain connectivity. First, due to volume conduction, the electrical potentials generated by neuronal activity are not only measured in the direct vicinity of neuronal sources but can also be measured at distant sites, substantially limiting the interpretation of between-channel synchronization measured by, e.g., coherence of phase locking. The authors suggested that one effective strategy for addressing this confounding factor is to confine the analysis to noninstantaneous correlations, e.g., by looking at the phase-lagged part of coherence. In the present study, we analyzed phase-lagged connectivity using a recent method characterized by improved sensitivity [41]. Second, Siegel et al. note that differences in measured correlations can be driven by differences in the signal-to-noise ratio (SNR). Even if the true correlation between two brain sources does not differ between experimental groups, differences in the signal amplitude alone lead to differences in the SNR and thus to differences in the connectivity measures. To exclude this confounding factor, we controlled for differences in signal amplitude between the groups. Third, a large number of interactions between regions/electrodes imposes the problem of multiple comparisons. To circumvent this problem, we applied the method for statistical analysis on large networks—network-based statistics (NBS) [42].

## Methods

### Participants and clinical assessment

This study is a part of the British Autism Study of Infant Siblings (BASIS), a UK collaborative network facilitating research with infants at risk for autism (http://www.basisnetwork.org) [43]. The study involved infants at high (HR) and low (LR) familial risk for ASD. Recruitment, ethical approval (UK National Health Service National Research Ethics Service London REC 08/H0718/76), and informed consent, as well as background data on participating families, were made available for the current study through the BASIS. The recruitment and diagnostic procedures, as well as attrition rate, are described in Additional file 2. EEG recording was performed when infants were on average 14 months old (range 12–17 months). Sufficient artifact-free EEG was available for 28 HR and 26 LR participants. At 36 months, a battery of clinical research measures was administered to HR participants (see Additional file 2: Table S2 for details). From the 28 HR infants, 10 were diagnosed with ASD at 3 years of age (HR-ASD). Demographic characteristics of the sample are given in Table 1. See Additional file 2 for details on participants’ enrollment, attrition rate, and details of the diagnostic assessment. The investigators involved in EEG processing and behavioral coding were blind to the outcome of the HR participants up to the final stage of data analysis.

**Table 1:** **Characteristics of the sample**

	LR	HR-no-ASD	HR-ASD
Number of male/female	12/14	3/15**	7/3
Age at EEG, in months	14.7 (1.2)	14.3 (1.7)	14.4 (1.3)
Age at diagnostic assessment, in months		37.5 (4.8)	38.6 (2.0)
Mullen Early Learning Composite, 14 months^a^	104 (17)**	100 (12)*	86 (15)
Mullen Early Learning Composite, 36 months^a^	115 (14)*	110 (18)	100 (27)

Notes: Means and standard deviations (in parentheses) are given for the ages and the Mullen ELC; difference between HR-ASD and comparison groups: *P <0.05, **P <0.01.

^a^Mullen was available for all but 1 of 26 LR infants at 14 months and 1 of 10 HR-ASD children at 36 months.

### Experimental procedure

The infants sat on their parents’ laps at a 60-cm distance from a 40 × 29 cm CRT monitor. Continuous EEG was sampled while participants watched three types of video stimuli, each lasting for 30–40 s: (1) a woman singing nursery rhymes or playing peek-a-boo (‘social’ video), (2) brightly colored toys moving and producing sounds (‘nonsocial’ video), and (3) the same sounding toys manipulated by a human hand (‘nonsocial’ video). Three triplets of video stimuli were presented in random order within the triplet (1-2-3, 2-3-1, etc.), but constant across the triplets for each participant. This resulted in nine 30–40-s EEG segments. Infants’ behavior during EEG session was recorded with a video camera.

### Behavioral analysis

Infants’ behavior and events interfering with attention to the experimental stimuli were coded off-line. The data from LR and HR babies were coded in a pseudo-random order. The following parameters were coded: (1) looking at the screen; (2) gross body, head, or arm movements; (3) crying; (4) smiling; and (5) interference from a parent, experimenter, or an activity interfering with movie watching (e.g., eating, sucking a pacifier). The infant was rated as attending to the video when she/he looked at the screen, did not move, and was not distressed. To assess inter-coder reliability, videos of five randomly chosen HR and five randomly chosen LR participants were double-coded by another researcher. High reliability was obtained for all variables (Spearman *rho*: looking 0.89, movements 0.85, crying 0.98, smiling 0.92, attention 0.92, interference 0.96).

### EEG acquisition and preprocessing

EEG was recorded using a 128-electrode HydroCel Geodesic Sensor Net (EGI, Eugene, OR) with respect to the vertex and sampled at 500 Hz. Twelve ridge electrodes most often contaminated by artifacts were excluded from analysis resulting in a 116-electrode layout (see Additional file 2: Figure S2). Data preprocessing and analysis was performed using FieldTrip (http://fieldtrip.fcdonders.nl/) as well as in-house software. The behavioral coding results were synchronized with EEG, and the periods when the baby was not looking at the screen, performed gross body, heard, or arm movements, or cried, as well as the periods of interference, were excluded from analysis. EEG was visually inspected for artifacts. The average length of the usable uninterrupted data periods (i.e., periods of artifact-free EEG corresponding to attentiveness in the absence of interference) did not differ between the HR-ASD and control groups (HR-ASD: 3.9 s, HR-no-ASD: 3.8 s, LR: 3.1 s; HR-ASD vs. LR and HR-ASD vs. HR-no-ASD: Mann-Whitney *U*-test *p*’s > 0.1). Each of the uninterrupted data periods was then segmented into 1-s segments with 50% overlap starting from the beginning of each clean EEG segment. The end part of the period shorter than one epoch was not analyzed. Neither number of included segments (minimum 120, see Additional file 2: Figure S1 for details) nor percent of interpolated data differed between HR-ASD and comparison groups (LR, HR-no-ASD; all *T*’s < 1.0, *P*’s > 0.33). See Additional file 2 for the preprocessing details.

### EEG power and connectivity analysis

Fast Fourier transforms (FFTs) were computed for each 1-s segment after removal of the mean (baseline correction) and application of the Hanning window. Power spectra were calculated as the magnitude-squared FFTs averaged across segments.

EEG connectivity can be strongly inflated by volume conduction [44]. Newly developed methods for assessment of phase-lagged connectivity allow us to avoid this problem by analyzing phase-shifted interactions. The advantage of this approach is that the presence of a consistent, nonzero phase lag between two time series cannot be explained by volume conduction from a single strong source and therefore reveals true interactions between different underlying generators. Phase relations between time series *X* and *Y* can be estimated by calculating their cross-spectrum for each of *N* data epochs. The negative value of the imaginary part (IP) of the cross-spectrum is equivalent of the signal *X* lagging signal *Y* in phase, while the opposite is true for the positive IP values. A few metrics have been suggested that quantify consistency of phase lag across data epochs or time points [41, 45–47]. In the present study, we used debiased weighted phase lag index (dbWPLI) [41] (see Additional file 2 for details). The significant advantages of this method over the other phase-lagged connectivity methods include its negligibly small sampling bias and improved capacity to detect true phase synchronization [41]. The dbWPLI values close to zero indicate absence of phase-lagged coupling while ‘1’ corresponds to the strongest possible coupling.

### Statistical analysis

Alpha power (7–8 Hz) was log-transformed prior to statistical analysis in order to normalize the distribution. We analyzed group differences in grand average alpha power, as well as the power of posterior alpha and central mu rhythms (see Additional file 2: Figure S2). A NBS [42] was used to compare network differences between HR-ASD and comparison groups (LR, HR-no-ASD), in order to control for the family-wise error rate (FWER) when testing is performed at each of the 6,670 connections. The primary cluster-defining threshold (Mann-Whitney *U*-test: *P* < 0.05, one-tailed, *Z* > 1.96) was first used to identify supra-threshold connections, within which the size (i.e., number of edges) of any connected components was then determined. A corrected *P* value was calculated for each component using the null distribution of maximal connected component size, which was derived empirically using a nonparametric permutation approach (5,000 permutations, *P* < 0.05). In addition, we calculated global alpha connectivity as an average dbWPLI over all possible pairs of connections. In cases where the distributions of connectivity values were not Gaussian (Shapiro-Wilk, *P* < 0.05), nonparametric tests were used to evaluate correlations and group differences.

## Results

### Behavioral analysis

The summary of behavioral results across the whole session, as well as separately for social and nonsocial videos (see Experimental procedure), is given in Additional file 2: Tables S3–S5. There were no significant differences between HR-ASD and the comparison groups in percent of time spent (1) looking at the videos, (2) moving, (3) watching the video without movement or negative affect (i.e., *attending* to videos), or (4) displaying affect (cry, smiles). To examine the possibility that infants from HR-ASD and the comparison groups preferred to attend to different types of stimuli (social vs. nonsocial), we tested for an effect of video on the infant’s attention using a repeated measures ANOVA with factors Stimulus-Type and Group. No violation of ANOVA assumption about homogeneity of variance was detected (Box’s *M* statistics, Levene’s test, *P* > 0.05). There was a main effect of Stimulus-Type (*F*_(1,53)_ = 91.2, *P* < 0.0001), indicating greater attention to social than nonsocial videos (percent of time when infants attended to social stimuli: mean = 91.1, sd = 9.6; nonsocial: mean = 74.0, sd = 14.6), but no effect of Group (*F*_(2,51)_ = 0.36, *P* = 0.7) or Group × Stimulus-Type interaction (*F*_(2,51)_ = 1.3, *P* = 0.28).

### EEG alpha power

All groups demonstrated occipital alpha and central mu spectral power peaks within infant alpha range [48], with a maximum at 7–8 Hz. No group differences in grand average alpha power, posterior alpha power, or mu power were found (see Additional file 2: Figures S2–S4 for details).

### The dbWPLI spectrum and distribution

**Figure 1:**
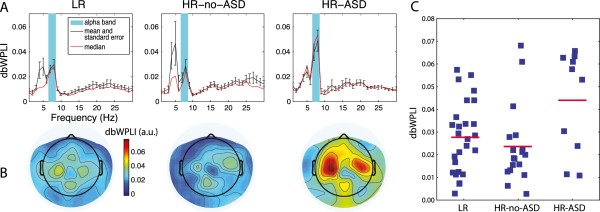
**Phase-lagged connectivity in the three groups of participants: LR (** ***N*** **= 26), HR-no-ASD (** ***N*** **= 18), HR-ASD (** ***N*** **= 10). (A)** Global connectivity as a function of frequency. Black lines show group means with vertical bars denoting standard errors; red lines show group median values. Cyan bars mark 7–8 Hz alpha range. **(B)** Scalp distributions of alpha-range dbWPLI values averaged for each electrode across all its connections (115 for each electrode). **(C)** Group means (red lines) and variability of global alpha connectivity.

In all experimental groups, the median values of global connectivity reached maximum at 7–8 Hz (Figure 1A), suggesting that the major part of interregional coupling during audiovisual attention occurred within the infant alpha band [48]. The other peak of grand average dbWPLI was observed in the theta range (4–5 Hz). The median value in the 4–5 Hz band (median = 0.015, range: 0.001–0.232) was lower than that in the 7–8 Hz band (median = 0.024, range: 0.003–0.068). We further limited the analysis to connectivity in the 7–8 Hz frequency range and provided the results for the theta in Additional file 2.

The choice of the narrow 7–8-Hz alpha band was justified by a sharp drop in global connectivity at either higher or lower frequencies in all groups of subjects (Figure 1A). Importantly, this frequency range largely escapes contamination by myogenic activity from the cranial muscles, which represent a major problem for analysis of ongoing high-frequency oscillations [38]. Figure 1B shows the scalp distribution of alpha dbWPLI values averaged for each electrode across all possible connections.

### Group differences in alpha connectivity

**Figure 2:**
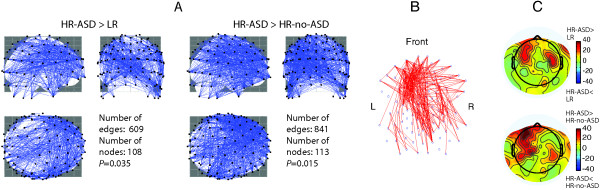
**Group differences in alpha-range connectivity between HR-ASD (** ***N*** **= 10) and comparison groups (LR,** ***N*** **= 26; HR-no-ASD,** ***N*** **= 18). (A)** Networks of increased connections in infants with ASD. NBS showed significantly higher functional connectivity in HR-ASD infants as compared to both LR and HR-no-ASD infants. The nodes (electrodes) and edges of the hyper-connected networks are loosely modeled on the standard brain image. **(B)** Overlap of the NBS clusters of the elevated connections revealed by HR-ASD vs. LR and HR-ASD vs. HR-no-ASD comparisons. **(C)** Difference between numbers of connections elevated (Mann-Whitney, *P* < 0.05, uncorrected) in the HR-ASD group and in comparison groups. Positive values correspond to a greater number of elevated connections in HR-ASD infants than in the comparison groups. Negative values correspond to a greater number of elevated connections in the comparison groups. Note clustering of over-connected sites over fronto-central regions.

For the HR-ASD vs. LR group comparison, the NBS identified one widespread pairwise cluster of hyper-connected regions in the HR-ASD group (*N* of nodes = 108, *N* of edges = 609, *P* = 0.035, Figure 2A; see also Additional file 2: Figure S5). A similar result was obtained for the HR-ASD vs. HR-no-ASD comparison (*N* of nodes = 113, *N* of edges = 841, *P* = 0.015, Figure 2A). When the analysis was repeated separately for the ‘social’ and ‘nonsocial’ videos, similar NBS results were obtained, suggesting that alpha hyper-connectivity occurs in infants that go on to ASD regardless of the type of video presented (see Additional file 2: Figure S6). The connections that were elevated in infants with ASD in relation to both LR and HR-no-ASD infants are plotted in Figure 2B. The electrodes with the greatest number of elevated connections were located in left fronto-central and right fronto-centro-temporal regions (Figure 2C).

The group means (LR: 0.0279, sd = 0.0157; HR-no-ASD: 0.0237, sd = 0.0173; HR-ASD: 0.0440; sd = 0.0224) and individual global connectivity values are shown in Figure 1C. The non-Gaussian distribution of global connectivity values was observed in the HR-ASD (Shapiro-Wilk *W* = 0.82, *P* = 0.026) and HR-no-ASD (Shapiro-Wilk *W* = 0.82, *P* = 0.003) groups. Therefore, the nonparametric Mann-Whitney *U*-test was applied to test for group differences in global connectivity. In line with the NBS results, the global alpha connectivity was also significantly elevated in the HR-ASD infants (HR-ASD vs. LR: *U*_(10,26)_ = 73, *Z* = 2.0, exact two-tailed *P* = 0.045; HR-ASD vs. HR-no-ASD: *U*_(10,18)_ = 48, *Z* = 1.99, exact two-tailed *P* = 0.045). Figure 1C shows that the increase of global connectivity in the HR-ASD group was driven by six of the ten infants.

The additional analysis suggested that group differences in connectivity were not dominated by connections within the right hemisphere or left hemisphere or by inter-hemispheric connections (Additional file 2: Figure S7). Moreover, the average connectivity calculated in the HR-ASD group separately for each hemisphere did not differ between hemispheres. However, there was a tendency for a higher connectivity in the left than in the right anterior area in participants with later ASD (see Additional file 2: Figure S8).

To test if the differences in connectivity between HR-ASD and the comparison groups could be explained by differences in gender or developmental level (Table 1), we analyzed effects of these variables separately in each experimental group. The absence of significant effects of these factors on connectivity (Additional file 2: Tables S7 and S8) makes such a possibility unlikely. There was no correlation between global connectivity and age in either of the groups (Additional file 2: Figure S10).

### Group differences in short- and long-range connectivity

**Figure 3:**
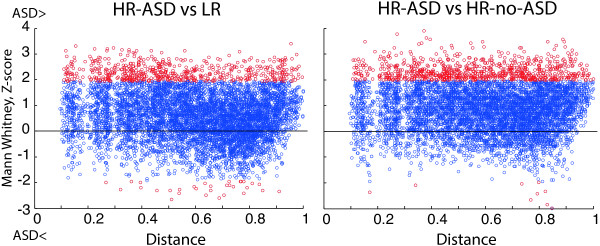
**Probability of group differences as a function of between-electrode distance.** *Ordinate*: *Z*-scores, Mann-Whitney *U*-test; *abscissa*: Euclidian distance between electrodes, where 1 corresponds to the maximal possible distance. Each pair of electrodes is represented by a dot. Red dots show *Z* values higher or lower then 1.96 (*P* < 0.05, two-tailed). For both HR-ASD (*N* = 10) vs. LR (*N* = 26) (*left panel*) and HR-ASD (*N* = 18) vs. HR-no-ASD (*N* = 18) (*right panel*) comparisons, the increased connectivity values in the HR-ASD group are observed irrespective of inter-electrode distance.

It has been previously suggested that children with ASD have a decreased long-to-short EEG connectivity ratio [49]. To check whether the hyper-connectivity in the HR-ASD group was due to short- or long-range connections, we plotted the probability of group differences in connectivity (*Z*-scores, Mann-Whitney *U*-test) as a function of between-electrode Euclidian distance (Figure 3). Inspection of the plots indicates that increased connectivity in the HR-ASD group does not depend on scalp electrode distances (see also Additional file 2: Figure S10).

### Alpha connectivity and severity of autism traits

**Figure 4:**
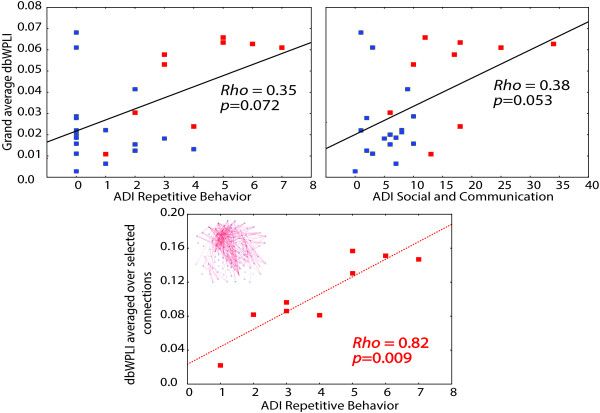
**Correlations (Spearman** ***rho*** **) between alpha-range connectivity and ADI-R domains in HR-ASD infants (** ***N*** = 9*) and combined HR group ( ***N*** = 26*). Red and blue squares mark, respectively, HR-ASD and HR-no-ASD infants. Global connectivity (*upper panels*) marginally correlates with the ADI-R Social and Communication composite score and with the ADI Repetitive Behavior score in the combined HR sample. The *lower panel* shows the correlation between the ADI-R Repetitive Behavior score and dbWPLI values averaged across connections that were significantly elevated in HR-ASD comparative to both comparison groups. *Note that one HR-ASD infant and one HR-no-ASD infant did not have ADI-R data.

In the whole sample of HR infants, there was a trend correlation between global connectivity and the Autism Diagnostic Interview-Revised (ADI-R) Restricted and Repetitive Behaviors (RRB) scale (Spearman *rho* = 0.35, *N* = 26, *P* = 0.072), as well as with a composite (arithmetic sum) of ADI-R Social and Communication scales (Spearman *rho* = 0.38, *N* = 26, *P* = 0.053) (Figure 4, *upper panel*) assessed at 3 years. No significant correlations with the Autism Diagnostic Observation Schedule (ADOS) Social Communication Total score or RRB score were found (*P*’s > 0.35). We further checked if the alpha-range connectivity in the regions that were hyper-connected in the HR-ASD infants was related to the severity of autism symptoms within the HR-ASD group. To do so, we calculated Spearman’s rank correlations between ADI-R/ADOS scales and dbWPLI averaged across all connections that reliably differentiated the HR-ASD from *both* comparison groups (Figure 2C). A significant correlation was found for the ADI-R RRB score (Spearman *rho* = 0.81, *N* = 9, *P* = 0.009, Figure 4, *lower panel*), but not for the ADI Social and Communication composite score (Spearman *rho* = 0.36, *P* = 0.33) or for ADOS domains (*P*’s > 0.35). No correlation with ADI-RRB has been found in the HR-no-ASD group, although this can be due to low variability of ADI-RRB scores in this group (the scores ranged 1–7 in the HR-ASD infants, while only two infants in the HR-no-ASD group had the ADI-RRB score over 2).

## Discussion

We observed increased phase-lagged alpha EEG connectivity in a group of 14-month-old infants who were later diagnosed with ASD at 3 years of age. The elevated connectivity was present despite the lack of measurable differences in behavior during the EEG data collection or differences in EEG spectral power. Further, in participants with ASD, this measure at 14 months correlated with the severity of restricted and repetitive behaviors at 3 years.

Studies in adults show a correlation between alpha EEG coherence and the structural integrity of white matter [50]. The high alpha-range connectivity in HR-ASD infants in our study may reflect the early maturation of white matter tracts previously reported in toddlers and young children with ASD [11–14]. The increases in white matter in ASD appear to reduce in toddlerhood [14] or later childhood [51, 52], finally resulting in predominantly hypo-connectivity between cortical areas in adults with ASD [7]. These age-related differences in white matter integrity may potentially explain the fact that the alpha-range hyper-connectivity detected in infants who later go on to have ASD in our study is later followed by alpha-range hypo-connectivity in adolescence and adulthood [31, 39, 53–56].

It is also possible that the atypical connectivity observed in ASD is a sequela of an altered excitation/inhibition (E/I) ratio [57]. The number of parvalbumin-expressing inhibitory interneurons [58], as well as GABA receptors and the receptors’ benzodiazepine binding sites [59], is reduced in autism, suggesting reduced cortical inhibition. In typical individuals, the glutamate/GABA ratio correlates positively with functional connectivity in the default mode network [60]. It is therefore possible that the elevated connectivity that we observed in HR-ASD infants results from their elevated E/I ratio. The later decrease in connectivity in adolescents and adults with ASD may then reflect a compensatory reaction to reduce cortical excitability. The future studies may help to investigate the putative link between abnormal neural connectivity and elevated E/I ratio in ASD by looking at connectivity measures in relation to ongoing and event-related gamma oscillations [61, 62].

The increased alpha-range connectivity in HR-ASD infants was widespread across the scalp (Figures 2A and 3), a finding that is in accord with DTI evidence on the presence of atypical development trajectories for white matter in all of the major tracts studied in toddlers with high autism traits [14] and with recent fMRI findings in children with ASD [8]. Nevertheless, clustering of significant dbWPLI differences over the anterior, and particularly left frontal (Figure 2B, C, see also Additional file 2: Figure S8), regions implies some degree of regional specificity in the atypical functional connectivity and suggests that the left frontal cortex may be particularly strongly over-connected in some infants who later go on to an ASD diagnosis. Prominent developmental atypicalities have previously been reported in the frontal lobes of children and adults with ASD [15, 16, 63]. Interestingly, Bashat and colleagues [11, 13] found abnormally mature white matter predominantly in the left frontal lobe in preschool children with ASD.

Alpha-range connectivity in infants who went on to ASD correlated with their later severity of RRB (Figure 4, *lower panel*). This finding suggests that alpha hyper-connectivity may reflect an important aspect of atypical brain function in many cases of emerging ASD. The link between hyper-connectivity and severity of RRB has previously been reported in resting-state fMRI studies. Uddin et al. [9] observed that hyper-connectivity in the ‘salience network’ predicted greater incidence of RRB in 10-year-old children with ASD. Delmonte et al. [64] observed a link between the severity of RRB and elevated fronto-striatal connectivity in adolescents with ASD. Interestingly, correlations between structural changes in the striatum and repetitive behaviors have been previously reported [65, 66] and may be secondary to changes in frontal areas or fronto-striatal connectivity [65]. In adults with ASD, the higher functional connectivity between left dorsal ACC and the frontal eye field was associated with more severe RRB, even though connectivity between these structures was reduced in the ASD participants relative to neurotypical controls [67]. A recent morphological study of short-range intrinsic cortico-cortical connections also reports an association between frontal connectivity and the ADI-R RRB score in adults with ASD [68]. Specifically, the decreased ‘wiring cost’ (and thus potentially increased cortico-cortical connectivity) in several frontal regions in participants with ASD correlated with their tendency to engage in repetitive behaviors. Although the sources of the elevated alpha connectivity in our study cannot be localized precisely based exclusively on surface EEG, our results accord well with the previous studies implicating frontal hyper-connectivity in repetitive behaviors in ASD.

It should be noted that *reduced* connectivity in young children with ASD has also been previously reported [69]. Using fMRI, Dinstein et al. [69] found that toddlers with ASD had reduced synchronization between corresponding ‘language areas’ of the two hemispheres during sleep. Moreover, the reduced functional connectivity correlated with poorer language abilities and with greater social and communication deficits. Further studies are therefore needed to investigate in ASD possible differences in functional connectivity measured at faster (EEG phase lag) and slower (fMRI) timescales, as well as modulations of connectivity by participant’s age and functional state.

It has been suggested that in adults with ASD, local hyper-connectivity in the frontal cortex occurs together with long-range hypo-connectivity [7, 16]. We have found elevated connectivity in infants with later ASD irrespective of between-electrode distance (Figure 3). Notably, the phase-lagged connectivity assessed in the present study may underestimate short-range connections as they are more likely than the long-range connections to occur without measurable phase lag. Other methods, such as, for example, alpha to gamma phase-amplitude coupling, may provide better estimates of short-range connectivity in ASD [54].

Although no EEG connectivity studies have been done so far in infants with later diagnosis of ASD, such studies have been previously performed in diagnosed children and adults. The studies in adults and adolescents with ASD mainly reported decreased MEG and EEG connectivity in the alpha frequency range [31, 39, 53–56], although negative findings have also been reported [70, 71]. Studies in children produced more variable results, with findings of decreased [72, 73], not changed [49, 74], both decreased and increased [75], or increased [76] alpha-range connectivity in ASD. Apart from differences in experimental samples, there are other factors that might potentially contribute to the findings. Among these are the choice of frequency band and reference electrode for EEG recording (e.g., Common Mode Sense-Driven Right Leg reference [74], linked earlobes [73, 76], Laplacian [75], or average reference [72]), presence of group differences in alpha power (and SNR) [72, 76] or lack of information on such differences [49, 74, 75], and differences in experimental paradigms, such as undefined state [49], rest with eyes closed [73], photo-driving [76], or visual stimulation with long (seconds) intervals [72, 74]. Some of these factors could influence group differences in alpha-range connectivity in our study. Moreover, the majority of the previous studies in children assessed functional connectivity using coherence—the measure that is sensitive to volume conduction. The application of the phase-lagged connectivity in our study should favor detection of the ‘true’ functional connectivity differences that could otherwise have been altered or canceled out by volume conduction effects that might be stronger in individuals possessing greater alpha power.

Although EEG and MEG have been previously used to study functional connectivity in ASD (see Additional file 1 for review), only one study, similarly to our present study, analyzed phase-lagged connectivity [74]. Boersma and colleagues recorded EEG in children (mean age 3 years) during presentation of visual stimuli but did not find group differences in low-frequency (4–10 Hz) connectivity. One possibility is that the elevated alpha range connectivity in ASD is more prominent during infancy and then decreases to the third year of life. Another possibility is that connectivity differences can still be detected in 3-year-olds with ASD but in narrower age-appropriate frequency bands and/or with a greater temporal quantity of data.

Figure 2C suggests that not all participants who were later diagnosed with ASD in our study had increased alpha-range connectivity as infants. Alterations in functional brain connectivity in ASD may depend on the presence of a risk allele of a particular ‘connectivity-crucial’ gene [77, 78] or on a combination of genes unique to an individual. Possibly, the atypical connectivity patterns characterize only a subset of the HR-ASD infants with such genetic predisposition. Our findings indicate that hyper-connectivity during infancy may be a feature of participants who later go on to show higher levels of restrictive and repetitive behavior (Figure 4). Considering the small group size of the HR-ASD infants in our study, further studies with larger samples are needed to elucidate behavioral features that may differentiate children with and without early alpha-range hyper-connectivity.

## Conclusions

In summary, our study provides the first EEG evidence of functional brain hyper-connectivity in infants who later go on to ASD. The hyper-connectivity may reflect morphological white matter abnormalities previously found in infants, toddlers, and young children with ASD and/or an elevated neural excitation/inhibition ratio. Future studies with participants of wider age ranges using robust EEG connectivity measures in combination with other neuroimaging techniques will help understand better the developmental course of neural connectivity in ASD and help provide neurophysiological biomarkers of these disorders.

## Electronic supplementary material

Additional file 1: **Short summary of the previous EEG and MEG studies of brain functional connectivity in ASD.** The file contains a table summarizing the main results of the studies and the methods used in these studies. (PDF 172 KB)

Additional file 2: **Supplementary methods and results.** Supporting methods: participants and clinical assessment; EEG recording and preprocessing; EEG connectivity analysis; minimal number of artifact-free EEG epochs included into analysis. Supporting results and discussion: behavioral analysis; theta band findings; power analysis of alpha activity; group differences in alpha ubPLI (combined conditions): NBS results; group differences in alpha dbWPLI in social and non-social conditions: NBS results; lateralization of alpha connectivity measures; effect of gender and developmental level; global alpha connectivity and age; dbWPLI group differences in short- and long-range connections in the alpha band. Supporting references. (DOCX 8 MB)

## Abbreviations

ASD: autism spectrum disorder (s)
dbWPLI: debiased weighted phase lag index
EEG: electroencephalography
FFT: fast Fourier transform
HR-ASD: high-risk participants with ASD outcome
HR: high risk
HR-no-ASD: high-risk participants with ‘no ASD’ outcome
LR: low risk
RRB: restricted and repetitive behaviors.
