# Supplementary material for: EEG hyper-connectivity in high-risk infants is associated with later autism
Source: J Neurodev Disord. 2014 Nov 7;6(1):40. doi: 10.1186/1866-1955-6-40 (PMC4232695; doi:10.1186/1866-1955-6-40)
Supplement: Supplementary file 1 — Additional file 1: Short summary of the previous EEG and MEG studies of brain functional connectivity in ASD. The file contains a table summarizing the main results of the studies and the methods used in these studies. (PDF 172 KB) [file 11689_2014_91_MOESM1_ESM.pdf]

Title
EEG hyper-connectivity in high-risk infants is associated with later autism

Authors
Orekhova E.V, Elsabbagh, M, Jones, E., Dawson, G., Charman, T., Johnson, M.H. and the BASIS team.

ADDITIONAL INTORMATION:

Summary of the previous EEG and MEG studies of brain functional connectivity in ASD.

*Abbreviation: ; **ACC** – anterior cingulate cortex; **AVE** – average reference; **CMS-DRL** - Common Mode Sense-Driven Right Leg reference; **IFG –** inferior frontal gyrus; **MI** - mutual information; **nvIQ*** – non-verbal IQ; ***PDC** - Partial directed coherence; **PLI** - phase lag index; **REF** – reference; **RIM** - robust interdependence measure; **SL -** Synchronization Likelihood.*

Authors/ Year	Age, Number of participants IQ	Method of connectivity analysis, EEG reference	Condition	Spectral power results	Connectivity results
Boersma et al. 2013 (1)	***3.5 yrs***<br>12 ASD,<br>IQ =108(12);<br>19 TD,<br>IQ =85(17)	EEG: ***PLI;***<br>REF: CMS-DRL	Passively watched pictures of cars and faces; trials of 1 s length (including 100 ms pre-stimulus)	Not analyzed	***No differences*** in connectivity strength in 4–10 Hz band; ***decreased connectivity*** in ASD in 10-25 Hz band.<br>*Graph analysis:* decreased 'small-worldness' in ASD at 4–10 Hz; Decreased clustering and increased path length at 10-25 Hz.

Kikuchi et al. 2014 (2)	***3.2-7.7 yrs*** 50 ASD 50 TD	MEG: ***Coherence*** between sensors located ~20 cm apart from one another	Watched a video program (with sound)	No differences	***Decreased*** theta coherence (with maximal effect at 6 Hz) between left frontal and right posterior sensors.
Isler et al, 2010 (3)	***5.5–8.5 yrs*** 6 ASD, IQ=67(27) 8 TD, IQ=117(10)	EEG: ***Coherence, Phase synchrony,*** REF: AVE	Stroboscopic flashes; ISI 1 sec; trials of 1 s length (100 ms pre-stimulus)	Power was increased in ASD	***Decreased*** inter-hemispheric occipital connectivity in ASD in wide frequency range (2-40 Hz)
Coben et al., 2008 (4)	***6-11 yrs*** 20 ASD, IQ=107(27.4); 20TD, IQ=110 (14.7)	EEG: ***Coherence;*** REF: Linked ears	Resting with eyes closed	ASD: Decreased absolute delta (1.5–3.5 Hz), and beta (12.5–25 Hz); enhanced relative theta (3.5–7.5 Hz); no differences in alpha (7.5–12.5 Hz)	***Decreased*** coherencies in delta, theta, alpha and beta bands.
Peters, et al., 2013 (5)	***0.08-25.6 yrs*** Tuberous Sclerosis patients with (14) and without ASD	EEG: ***Coherence,*** REF: AVE	?	Not analyzed	***No coherence differences*** in 4 - 8Hz, 8-10Hz and 10-12Hz bands; decreased long over short range coherence ratio in ASD. *Graph analysis*: No differences in network topography; increased

	(29), non-syndromic ASD (16), and controls (46). IQ?				resilience in ASD
Duffy et al., 2012 (6)	***2-12 years*** 430 ASD 554 TD IQ?	EEG: ***Coherence,*** REF: Laplacian	?	Not analyzed	1-32 Hz range in 2-Hz bands: ***decreased short-distance*** and ***decreased, as well as increased, long-distance*** coherences in ASD in various bands.
Lazarev et al., 2009 (7)	***6–16 yrs*** 14ASD IQ=91(22), 19TD IQ?	EEG: ***Coherence***; REF: unilateral references to the corresponding earlobe	Photic stimulation at 12 frequencies between 3–27 Hz and resting state	Decreased power in the right hemisphere at all driving frequencies	***Coherence increased*** in the left hemisphere in ASD at the EEG frequencies corresponding to stimulation frequencies (6–27 Hz). No connectivity differences under rest.
Velazquez et al., 2009 (8)	***7 -16 yrs***, 15 ASD, IQ=111.2(15); 16 TD, IQ=123(8)	MEG: ***Phase synchrony*** at 10, 18, 26 and 32 Hz	Stroop colour word task	Not analyzed	*Lack of task-related increase of coherence* in ASD;
Han et al., 2013 (9)	***8–17 yrs*** 17 HF-ASD IQ= 106 (20); 17 LF-ASD IQ= 56 (12) No TD control	EEG: ***Coherence*** REF: Linked ears	Resting with eyes open	Not analyzed	***Increased coherence*** in 4-8 Hz band ***in low-functioning ASD compared to high-functioning ASD***. Other bands were not analyzed

Lazar et al., 2010 (10)	***7.5–21.5 yrs*** 18 ASD 14 TD, matched on nvIQ (Raven)	EEG: ***Coherence***, REF: Linked mastoids	Non-REM sleep	Increased power in delta (0.5–4 Hz) and decreased in alpha (8–10.75 Hz), sigma (11-15 Hz), beta (15.25–25 Hz), gamma 1 (25.25–35 Hz) and gamma 2 (35.25–45 Hz) bands.	***Decreased coherences*** in delta, theta, alpha and sigma bands.
Khan et al., 2013 (11)	***13-21 yrs*** 17 ASD, nvIQ=112(18) 20 TD nvIQ=112(16)	MEG: ***Event-related coherence*** *in source space*	Pictures of houses and faces presented for 1 s with 1 s ISI	No power differences	***Decreased long-range coherence*** between fusiform face area and precuneus, IFG, and ACC in ASD, confined to the alpha band.<br>Decrease alpha-gamma phase-amplitude coupling, pointing to ***decreased local coherence***.<br>Correlations with ADOS social score
Tsiaras et al., 2012 (12)	**17–21 yrs** 8 ASD, IQ=107(14)	MEG: ***RIM, PDC, MI***	Supine, rest with eyes closed	Not analyzed	***Decreased short-range connections*** in ASD within bilateral temporal, frontal and left

	8 TD<br>IQ=123(6);				parietal sector. ***Decreased long-range connectivity*** of temporal and frontal regions with all other regions. Group differences were greatest for RIM and restricted to the alpha band.
Barttfeld et al., 2011 (13)	***Adults***<br>10 ASD<br>IQ=107(15)<br>10 TD<br>IQ?	EEG: ***SL***<br>REF: AVE	Rest, eyes-closed	Not analyzed	***Decreased long-range connectivity*** *in ASD*, most prominent for fronto-occipital connections. ***Increased short-range connectivity*** in lateral–frontal electrodes. Only delta range (0.5-3Hz) was analyzed. *Graph analysis*: decreased 'small-worldness' in ASD.
Barttfeld et al., 2013 (14)	***Adults***<br>74 neuro-typical subjects	EEG: ***SL;***<br>REF: AVE	Rest, eyes-closed	Not analyzed	***Decreased connectivity*** was associated with an increasing number of autistic traits.<br>The effect was greatest for in delta (0.5-4 Hz) and theta (4 Hz to 8 Hz) bands, was present for beta (15-25 Hz) and gamma (25-35 Hz) and was absent for alpha (8-12 Hz) and sigma (12 -15 Hz), bands .<br>*Graph analysis*: 'small-worldness' in the delta band was negatively related to amount of autistic

					traits.
Catarino et al., 2013 (15)	***Adults*** 15 ASD, IQ=119(14) 15 TD, IQ=119(13)	EEG: ***Wavelet coherence,*** REF: tip of the nose	Presentation of faces and chairs; matching tasks; 0-400 ms interval after stimulus onset	No differences	***Decreased inter-hemispheric coherence*** in ASD for all electrode pairs studied. Group differences were observed after 150 ms and at frequencies lower than 13 Hz
Kenet et al., 2012 (16)	***Adults*** 11 ASD IQ=116(8) 11TD IQ=113(9)	MEG: ***Coherence,*** *in source space*	Baseline, as well as periods of preparation for pro- or anti-saccades	No difference	***Decreased*** alpha band ***coherence*** in ocular motor control network in ASD. Absence of task-related increase in coherence in ASD.
Leveille et al., 2010 (17)	***Adults*** 9 ASD, IQ= 101.3, 11 TD, IQ=115.7	EEG: ***Coherence*** REF: Linked ears	REM sleep	Not analyzed	***Increased*** left intra-hemispheric ***coherence*** with 'O1' in ASD; ***decreased coherence*** between F6-F4. The differences were significant in delta (0.75–3.75 Hz), and theta (4.00–7.75 Hz), but not in alpha (8.00–12.75 Hz), sigma (12.00–14.00 Hz), or beta (13.00–20.25 Hz) bands. No differences for inter-hemispheric coherence
Mathewson et al., 2012 (18)	***Adults*** 15 ASD, IQ=107(12); 16 TD,	EEG, ***Coherence,*** REF: Cz	eyes-closed and eyes-open	No differences	Only alpha band (8–13 Hz) analyzed. ***No group differences in coherence.***

	101 (19)				
Murias et al., 2007 (19)	***Adults,*** 18 ASD, IQ>80; 18 TD, IQ?	EEG, ***Coherence,*** REF: AVE	Rest, eyes-closed	Theta (3-6 Hz) power was increased; alpha (8-10 Hz) power was decreased in ASD	***Coherence increased*** in theta and ***decreased*** in alpha band in ASD.

References

1. Boersma M*, et al.* (2013) Disrupted functional brain networks in autistic toddlers. *Brain connectivity* 3(1):41-49.
2. Kikuchi M*, et al.* (2014) Reduced long-range functional connectivity in young children with autism spectrum disorder. *Social cognitive and affective neuroscience*.
3. Isler JR, Martien KM, Grieve PG, Stark RI, & Herbert MR (2010) Reduced functional connectivity in visual evoked potentials in children with autism spectrum disorder. *Clin Neurophysiol* 121(12):2035-2043.
4. Coben R, Clarke AR, Hudspeth W, & Barry RJ (2008) EEG power and coherence in autistic spectrum disorder. *Clin Neurophysiol* 119(5):1002-1009.
5. Peters JM*, et al.* (2013) Brain functional networks in syndromic and non-syndromic autism: a graph theoretical study of EEG connectivity. *Bmc Med* 11.
6. Duffy FH & Als H (2012) A stable pattern of EEG spectral coherence distinguishes children with autism from neuro-typical controls - a large case control study. *Bmc Med* 10.
7. Lazarev VV, Pontes A, Mitrofanov AA, & deAzevedo LC (2010) Interhemispheric asymmetry in EEG photic driving coherence in childhood autism. *Clin Neurophysiol* 121(2):145-152.
8. Velazquez JLP*, et al.* (2009) Decreased brain coordinated activity in autism spectrum disorders during executive tasks: Reduced long-range synchronization in the fronto-parietal networks. *Int J Psychophysiol* 73(3):341-349.
9. Han YMY*, et al.* (2013) Altered immune function associated with disordered neural connectivity and executive dysfunctions: A neurophysiological study on children with autism spectrum disorders. *Res Autism Spect Dis* 7(6):662-674.
10. Lazar AS*, et al.* (2010) Reduced fronto-cortical brain connectivity during NREM sleep in Asperger syndrome: An EEG spectral and phase coherence study. *Clin Neurophysiol* 121(11):1844-1854.
11. Khan S*, et al.* (2013) Local and long-range functional connectivity is reduced in concert in autism spectrum disorders. *P Natl Acad Sci USA* 110(8):3107-3112.
12. Tsiaras V*, et al.* (2011) Extracting biomarkers of autism from MEG resting-state functional connectivity networks. *Computers in biology and medicine* 41(12):1166-1177.
13. Barttfeld P*, et al.* (2011) A big-world network in ASD: Dynamical connectivity analysis reflects a deficit in long-range connections and an excess of short-range connections. *Neuropsychologia* 49(2):254-263.

14. Barttfeld P*, et al.* (2013) Organization of brain networks governed by long-range connections index autistic traits in the general population. *J Neurodev Disord* 5.
15. Catarino A*, et al.* (2013) Task-related functional connectivity in autism spectrum conditions: an EEG study using wavelet transform coherence. *Molecular autism* 4(1):1.
16. Kenet T*, et al.* (2012) Disconnectivity of the cortical ocular motor control network in autism spectrum disorders. *Neuroimage* 61(4):1226-1234.
17. Leveille C*, et al.* (2010) Enhanced Connectivity Between Visual Cortex and Other Regions of the Brain in Autism: A REM Sleep EEG Coherence Study. *Autism Res* 3(5):280-285.
18. Mathewson KJ*, et al.* (2012) Regional EEG alpha power, coherence, and behavioral symptomatology in autism spectrum disorder. *Clin Neurophysiol* 123(9):1798-1809.
19. Murias M, Webb SJ, Greenson J, & Dawson G (2007) Resting state cortical connectivity reflected in EEG coherence in individuals with autism. *Biol Psychiat* 62(3):270-273.
