# Supplementary material for: EEG hyper-connectivity in high-risk infants is associated with later autism
Source: J Neurodev Disord. 2014 Nov 7;6(1):40. doi: 10.1186/1866-1955-6-40 (PMC4232695; doi:10.1186/1866-1955-6-40)
Supplement: Supplementary file 2 — Additional file 2: Supplementary methods and results. Supporting methods: participants and clinical assessment; EEG recording and preprocessing; EEG connectivity analysis; minimal number of artifact-free EEG epochs included into analysis. Supporting results and discussion: behavioral analysis; theta band findings; power analysis of alpha activity; group differences in alpha ubPLI (combined conditions): NBS results; group differences in alpha dbWPLI in social and non-social conditions: NBS results; lateralization of alpha connectivity measures; effect of gender and developmental level; global alpha connectivity and age; dbWPLI group differences in short- and long-range connections in the alpha band. Supporting references. (DOCX 8 MB) [file 11689_2014_91_MOESM2_ESM.docx]

**ADDITIONAL METHODS and RESULTS**

***Title***

**EEG hyper-connectivity in high-risk infants is associated with later autism**

***Authors***

Orekhova E.V, Elsabbagh, M, Jones, E., Dawson, G., Charman, T., Johnson, M.H. and the BASIS team.

**Supporting Methods**

***Participants and clinical assessment***

Recruitment, ethical approval (UK National Health Service National Research Ethics Service London REC 08/H0718/76), and informed consent, as well as background data on participating families, were made available for the current study through the British Autism Study of Infant Siblings (BASIS), a UK collaborative network facilitating research with infants at risk for autism (www.basisnetwork.org). Infants participating in the study had an older sibling with (high risk group; HR) or without (low risk group; LR) ASD. Families enrolled from various regions when their babies were younger than 5 months of age and they were invited to attend multiple research visits (6-9, 12-15, 24 and 36 months) until their children reach 3 years of age or beyond.

At the time of enrollment in the BASIS, none of the participating infants had been diagnosed with any major medical or developmental condition. Infants in the HR group all had an older sibling (hereafter, proband) with a community clinical diagnosis of an autism spectrum disorders (ASD; or in 4 cases, a half-sibling), and in 3 cases 2 probands with an ASD. 45 probands were male, 9 were female. Proband diagnosis was confirmed by two expert clinicians (PB, TC) based on information using the Development and Wellbeing Assessment (DAWBA)1 and the parent-report Social Communication Questionnaire (SCQ)2*.* Most probands met criteria for ASD on both the DAWBA and SCQ (n = 44). While a small number scored below threshold on the SCQ (n = 4) no exclusions were made, due to meeting threshold on the DAWBA and expert opinion. For 2 probands, data were only available for either the DAWBA (n = 1) or the SCQ (n=1). For 4 probands, neither measure was available (aside from parent-confirmed local clinical ASD diagnosis at intake). Parent-reported family medical histories were examined for significant medical conditions in the proband or extended family members, with no exclusions made on this basis.

Infants in the LR group were recruited from a volunteer database at the Birkbeck Centre for Brain and Cognitive Development. Inclusion criteria included full-term birth (with one exception), normal birth weight, and lack of any ASD within first-degree family members (as confirmed through parent interview regarding family medical history). All infants in the LR group had at least one older-sibling (in 3 cases, only half-sibling). 28 of the older siblings were male, 22 were female. Screening for possible ASD in these older siblings was undertaken using the SCQ, with no child scoring above instrument cut-off for ASD (>15) (one score was missing).

Alongside the standard measures of cognitive (Mullen Scales of Early Learning) ([1](#_ENREF_1)) and adaptive (Vineland Adaptive Behavior Scales) ([2](#_ENREF_2)) development taken at each visit, at 24 months (HR group only, 50 Module 1, 2 Module 2) and 36 months (both groups; 3 Module 1, 98 Module 2) a semi-structured play assessment, the Autism Diagnostic Observation Schedule (ADOS)- Generic ([3](#_ENREF_3)) was used to assess autism-related social and communication behavioral characteristics. This was augmented at 36 months (HR group only) with the parent-report Autism Diagnostic Interview-Revised (ADI-R) ([4](#_ENREF_4)).

For the HR group consensus ICD-10 ([5](#_ENREF_5)) ASD (including childhood autism, atypical autism, and other pervasive developmental disorder (PDD)) was diagnosed using all available information from all visits by experienced researchers. From the initial group of 53 toddlers assessed at 36-months, 17 (11 boys, 6 girls) met criteria for an ASD diagnosis (32.1%). Given the young age of the children, and in line with DSM-5 ([6](#_ENREF_6)), no attempt was made to assign specific subcategories of PDD/ASD diagnosis.

It is worth noting that the recurrence rate reported in the current study (32.1%) is higher than that reported in the large consortium paper published by Ozonoff et al. {Ozonoff, 2011 #276}(18.7%). This is likely to reflect the modest high-risk sample size in the current study, with accompanying error (n = 53). While recurrence rates approaching 30% have been found in other moderate size samples ({Paul, 2011 #277;Landa, 2006 #278}, these rates are sample specific and will likely not be generalizable, as findings from larger samples show autism recurrence rates converge between 10% and 20% {Ozonoff, 2011 #276;Constantino, 2010 #279;Sandin, 2014 #280}. Similar procedures combining all information from standard diagnostic measures and clinical observation and arriving at a clinical best estimate ICD-10 diagnosis were used in the present study in line with other familial at-risk studies and were conducted by an experienced group of clinical researchers.

One hundred and three infants from BASIS took part in the current EEG study (53 HR, and 48 LR) at the 12 to 15-month time-point. Data from thirteen infants were lost because of technical failures (6 HR and 7 LR). Two infants fell asleep during the EEG session (1 HR, 1 LR) and one participant in the HR group missed diagnostic assessment at the 36-month visit; data from these 3 infants were also excluded. From the rest of the subjects sufficient artifact-free EEG data were obtained in 26 LR and 28 HR infants.

The table *S2* presents ADI-R and ADOS scores for the three groups of participants.

*Table S2*. Mean (SD) ADI-R and ADOS scores at 3 years

|  | LR | HR-no-ASD | HR-ASD |
| --- | --- | --- | --- |
| ADI-R Social and Communication* | - | 5.3 (3.3)  N=17 | 17.0(8.4)  N=9 |
| ADI-R,  RRB† | - | 0.8 (1.2)  N=17 | 4.0 (1.9)  N=9 |
| ADOS,  Total | 6.0 (4.3) | 8.0 (5.7)  N=18 | 13.0 (4.8)  N=10 |
| ADOS,  RRB | 1.1 (1.1) | 0.7 (1.1)  N=18 | 2.8 (1.5)  N=10 |

* A composite (arithmetic sum) of Social and Communication scales

† Restricted and Repetitive Behaviors

*Note*: ADI-R data was available in 17 of 18 HR-no-ASD infants, in 9 of 10 HR-ASD infants and was not available in the LR group.

***EEG recording and preprocessing***

The EEG recordings were performed between 09:00 and 18:00, when the child was calm, but active. There was no difference in time of the experiment start between HR-ASD (mean=13.2 h, sd=1.96) and the LR (mean=12.5 h, sd=1.39; T_(34)_=1.3, *P*=0.19) or HR-no-ASD (mean=13.2 h, sd=1.91; T_(26)_=0.27, *P*=0.97) comparison groups.

Data preprocessing and analysis was performed using FieldTrip (http://fieldtrip.fcdonders.nl/) as well as in-house software. Twelve ridge electrodes most frequently contaminated by artifacts were excluded from analysis, resulting in a 116-electrode layout (Fig. S3). EEG was visually inspected for artifacts and bad channels were marked. The bad channels were interpolated by replacing them with the average of their neighbors (nearest-neighbor approach) and re-referenced to the average of all electrodes. The periods of EEG contaminated by motion artifacts affecting a group of channels or by blinks were marked and further rejected. The rest of the data were divided into 1-sec segments with 50% overlap. For each segment the channels with absolute amplitude exceeding 300 mkV or those with amplitude ‘jumps’ (>=100 mkV amplitude change during 4 ms or less) were interpolated. The overall maximal allowed number of interpolated channels for each segment was 17 (15%). The minimal number of segments included into analysis was 120.

***EEG connectivity analysis***

For two different channels *x* and *y*, the cross-spectrum was obtained by multiplying Fourier transformed signals *X*(*f*) and *Y*(*f*): *Z* ( *f* ) *= X*( *f* )*Y**( *f* ), where *Y** indicates the complex conjugate of *Y*. The PLI for channels *x* and *y* is defined as the absolute average [over epochs] value of the sign of the imaginary part of Z, $\mathfrak{I}\left\{ Z \right\}$ ([7](#_ENREF_7)):

$PLI= \left| E\{sgn(\mathfrak{I}\left\{ Z \right\})\} \right|.$ (1)

The PLI is positively biased, i.e. it is generally higher for a lower number of averaged epochs. The unbiased version of PLI (ubPLI) can be calculated as the average of all pairwise products of signs ([8](#_ENREF_8)):

$ubPLI= E\{sgn\left( \mathfrak{I}\left\{ Z_{j} \right\} \right)*sgn\left( \mathfrak{I}\left\{ Z_{k} \right\} \right)\}$ , (2)

where indexes *j* and *k* indicate different epochs. The weighted PLI (WPLI), that is less sensitive to noise than PLI ([8](#_ENREF_8)) is defined as:

$WPLI= \frac{\left| E\{\mathfrak{I}\left\{ Z \right\}\} \right|}{E\left\{ \left| \mathfrak{I}\left\{ Z \right\} \right| \right\}}$ . (3)

Similar to PLI the WPLI is positively biased. The debiased version of WPLI (dbWPLI) can be calculated as

$dbWPLI=\frac{\sum_{j=1}^{N} \sum_{k\neq j} \mathfrak{I\{}Z_{j}\mathfrak{\}I\{}Z_{k}\}}{\sum_{j=1}^{N} \sum_{k\neq j} \mathfrak{|I}\left\{ Z_{j} \right\}\mathfrak{I}\left\{ Z_{k} \right\}|}$, (4)

where the numerator is equal to the sum of all pairwise products of imaginary components and the denominator is equal to the sum of all pairwise products of the magnitudes of the imaginary components ([8](#_ENREF_8)). For small sample sizes dbWPLI is slightly negatively biased when WPLI exceeds the PLI, and positively biased when PLI exceeds the WPLI, but these biases are negligibly small for large number of averages ([8](#_ENREF_8)).

Тhe dbWPLI assumes statistical independence between analyzed epochs. Therefore, following the strategy applied by Vinck et al ([9](#_ENREF_9)) we excluded from the numerator and denominator in eq. (2, 4) pairs of epochs *Z_j_Z_k_* corresponding to the overlapping segments.

Similarly to the other phase lag measures the dbWPLI is robust to volume conduction. The other advantages of the dbWPLI are its negligibly small sampling bias and its improved capacity to detect true differences in phase synchronization due to its lower sensitivity to noise as compared to the previously suggested PLI ([7](#_ENREF_7)).

In order to check if weighting by the magnitude of the imaginary part of the cross-spectra could have an effect on group differences we also calculated the unweighted version of the phase lag index – unbiased phase lag index (ubPLI, *equation 2*) ([8](#_ENREF_8)). The results, although less reliable, were very similar to those obtained with the dbWPLI (*Fig. S4*).

***Minimal number of artifact-free EEG epochs included into analysis***

The dbWPLI values were calculated for all participants with at least 40 1-sec segments of artifact-free data. The initial minimum of 40 EEG data epochs has been chosen because the de-biased WPLI can be still biased in case of smaller amount of data (8).

Figure S1 shows individual global connectivity values as a function of number of EEG data epochs across all subjects. The only extremely high (HR-no-ASD infant; the value exceeds 3 SD) and the only negative (HR-no-ASD infant) global connectivity values were associated with small number of epochs, suggesting low reliability of connectivity estimation. We therefore further limited analysis to the subjects with more then 120 epochs (more then one minute) of artifact-free EEG data.


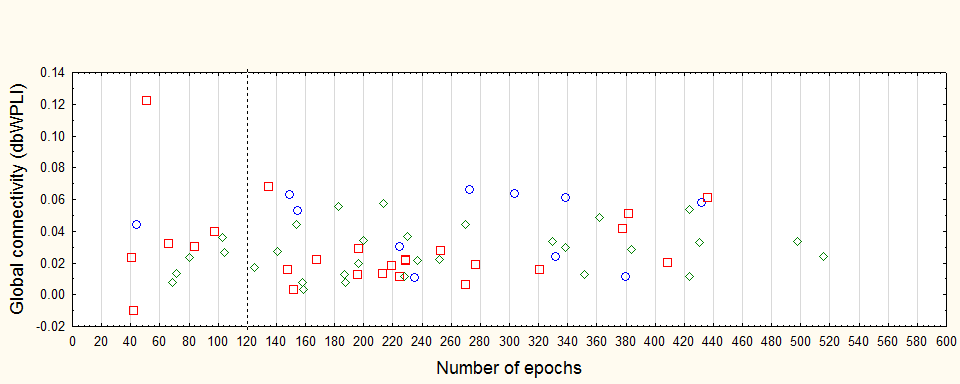


*Figure S1*. Grand average connectivity values as a function of number of EEG data epochs for all subjects (31 LR: green, 23 HR-no-ASD: red, 11 HR-ASD: blue) with number of artifact-free EEG epochs >= 40.

Importantly, inclusion of the subjects with fewer than 120 epochs did not significantly change between-group differences in global connectivity: HR-ASD vs LR: Mann-Whitney U_(11,31)_=90, Z=2.29, exact 2-*sided* *P*=0.020496; HR-ASD vs HR-no-ASD: U_(11,31)_=71, Z=2.02, exact 2-*sided* *P*=0.041590. Means (sd) for LR: 0.0268 (0.0151) ; HR-no-ASD: 0.0275 (0.0269); HR-ASD: 0.0440 (0.0213).

**Supporting Results and Discussion**

***Behavioral analysis***

T-test was used to analyze group differences in ‘Looking’, ‘Motion’, and ‘Attention’ behavior. The distributions of ‘Crying’ and ‘Smiling’ behaviors were significantly different from normal. Therefore, Mann-Whitney test was used in these cases.

*Table S3.* Results of behavioral analysis across conditions: percent of time when infants demonstrated the behavior and the probability of group differences. The mean (SD) is given for %Look, %Move and %Attend. The median (quartile range) is given for %Cry and %Smile, because these variables had non-normal distributions.

| Behavioral measure | LR  *N=26* | HR-no-ASD  *N=18* | HR-ASD  *N=10* |
| --- | --- | --- | --- |
| % Look | 92.8(6.2)  *p*=0.95* | 90.6(4.8)  *p=0.31* | 92.9 (7.0) |
| % Move | 16.3(10.8)  *p=0.49* | 17.6(8.9)  *p=0.27* | 13.6 (9.0) |
| % Smile | 0.0 (0.0)  *p=0.08* | 0.0 (1.33)  *p=0.27* | 0.0 (3.75) |
| % Cry | 1.93 (10.8)  *p=0.75* | 2.23 (4.23)  *p=0.72* | 0.5 (10.94) |
| % Attend | 78.5(13.6)  *p=0.65* | 78.3(9.2)  *p=0.55* | 80.8(12.4) |

*P-values show probability of difference between the HR-ASD and the comparison groups (LR or HR-no-ASD).

*Table S4.* Results of behavioral analysis for ‘social’ condition: mean (SD) percent of time when infants demonstrated the behavior and the probability of group differences. The mean (SD) is given for %Look, %Move and %Attend. The median (quartile range) is given for %Cry and %Smile, because these variables had non-normal distributions.

| Behavioral measure | LR  *N=26* | HR-no-ASD  *N=18* | HR-ASD  *N=10* |
| --- | --- | --- | --- |
| % Look | 97.5(3.2)  *p=0.16* | 97.6(4.4)  *p=0.34* | 99.0(1.6) |
| % Move | 9.1(9.3)  *p=0.14* | 5.8(6.9)  *p=0.59* | 4.4(5.5) |
| % Smile | 0.0 (0.0)  *p=0.06* | 0.0 (3.21)  *p=0.24* | 1.37 (8.12) |
| % Cry | 0.0 (4.18)  *p=0.10* | 0.0 (1.46)  *p=0.43* | 0.0 (0.0) |
| % Attend | 88.8(11.1)  *p=0.11* | 92.4(8.6)  *p=0.42* | 94.9(5.2) |

*Table S5.* Results of behavioral analysis for ‘non-social’ conditions: mean (SD) percent of time when infants demonstrated the behavior and the probability of group differences. The mean (SD) is given for %Look, %Move and %Attend. The median (quartile range) is given for %Cry and %Smile, because these variables had non-normal distributions.

| Behavioral measure | LR (N=26) | HR-noASD (N=18) | HR-ASD (N=10) |
| --- | --- | --- | --- |
| % Look | 91.1(7.6)  *p=0.86* | 87.9(6.5)  *p=0.38* | 90.5(9.5) |
| % Move | 19.0(12.3)  *p=0.66* | 22.5(12.3)  *p=0.26* | 17.0(11.7) |
| % Smile | 0.0(0.0)  *p=0.52* | 0.0 (0.0)  *p=0.50* | 0.0 (0.0) |
| % Cry | 2.67 (13.59)  *p=0.90* | 3.06 (5.99)  *p=0.79* | 0.69 (11.37) |
| % Attend | 74.6(15.4)  *p=0.92* | 72.5(12.6)  *p=0.64* | 75.2(17.1) |

***Theta band findings***

Distribution of theta power was strongly different from the Gaussian in social and combined conditions (Social: Shapiro-Wilk W=0.77, p=0.00000; Combined: Shapiro-Wilk W=0.87, p=0.00002). There was no difference in the grand average theta power between HR-ASD and control groups in social, non-social or combined conditions (Mann-Whitney U Test; all p’s>0.2). The distribution of grand average dbWPLI was strongly different from the Gaussian in all conditions (p<0.0001). No reliable group differences in dbWPLI were found using NBS (all p’s>0.1). The grand average dbWPLI did not differ between the HR-ASD and the control groups.

The absence of group differences in the theta band and presence of such differences in the alpha band (see the main manuscript) may indicate greater vulnerability of the alpha-generating circuits in ASD. On the other hand, a few considerations listed below suggest that the negative findings in the theta band have to be considered with caution.

*First,* the median values of the grand average dbWPLI were generally lower in the theta then in the alpha band, suggesting a less optimal signal-to-noise ratio.

*Second*, the theta is strongly modulated by cognitive effort and emotional state of the infant (Stroganova 2007). Although we controlled for subject’s attentiveness to the stimulation, we could not control for infant’s emotional or cognitive engagement during the passive viewing condition. These uncontrolled factors could strongly affect inter-individual variability in theta power (and connectivity) and obliterate the true group differences.

*Third*, neither posterior alpha, nor mu power significantly differed in social vs. non-social conditions (p>0.1). Therefore, in order to increase statistical power of the alpha-range connectivity analysis we combined the data across conditions. This is more problematic for theta, because theta power was strongly affected by condition (Sign test: Z=4.7, p=0.000003). As the result, the between-subjects differences in amount of data obtained from each of the conditions contributed to the between-subjects variability in theta power (and connectivity).

*Fourth,* distributions of posterior alpha and mu power did not differ from the Gaussian while the theta power had clearly non-Gaussian distribution in social and combined conditions. There were participants with very strong theta power (outliers) in all three groups. Inclusion of the power outliers might affect connectivity through e.g. affecting signal-to-noise ratio. On the other hand, their exclusion would decrease the sample size and statistical power.

We believe that the future studies of theta range connectivity in infants, including those with ASD have to apply well-control experimental conditions and take all the listed factors into consideration.

***Power analysis of alpha activity***

Differences in spectral power may potentially affect connectivity differences by affecting the signal to noise ratio. Therefore, we tested for possible differences in alpha power between the HR-ASD and the comparison groups using T-test. For grand average alpha power no group differences were detected (HR-ASD vs LR: T_(26)_=0.93, *P*=0.98; HR-ASD vs HR-no-ASD: T_(34)_=0.62, *P*=0.43; LR mean= 2.91, sd=0.51; LR-no-ASD mean=2.66; sd=0.40; HR-ASD mean=2.80, sd=0.39).

We also tested for differences in posterior alpha power and power of sensorimotor (mu) rhythm, separately in the left and right hemispheres. Power of these rhythms was calculated based on electrode selection shown in figure *S2*. Figure *S3(A)* shows the mean EEG power spectra for the electrode selections. Scalp distribution of alpha (7-8 Hz) spectral power is plotted in figure *S3(B)*. No group significant differences in posterior alpha and sensorimotor mu power were found (*P*’s>0.13).

*Figure S2*. EEG electrodes used to calculate spectral power of posterior alpha and sensorimotor (mu) rhythm.

*Figure S3*. Alpha power in three groups of participants. (A) Mean power spectra at anterior and posterior regions plotted in figure *S2*. Cyan bars mark alpha range (7-8 Hz). Note spectral peaks corresponding to posterior alpha (solid lines) and central mu (dashed lines) rhythms. (B) Scalp distribution of alpha power.

According to the literature, the amplitude of the sensorimotor (mu) rhythm is modulated by several factors. First, it is suppressed during the subject’s own motion {Kuhlman, 1978 #265}. Second, it increases during states of ‘hyper-vigilance’, such as intense attentiveness {Rougeul-Buser, 1975 #264}. Third, it decreases during observation of other’s movements (supposedly, reflecting mirror neuron functioning) {Oberman, 2007 #266}. All these factors differed between the types of videos presented in our study. Specifically, infants in all groups attended least and moved most during the ‘toy-without-hand’ condition; the ‘social’ condition produced most attention and least movements, while the ‘hand-with-toy condition’ was in between (Figures 1, 2 below). These differences predict the highest level of mu during the ‘social’ condition that was characterized by reduced motor activity and increased attentiveness. On the other hand, the presence of human motion in the ‘toy/hand’ and social videos might result in suppression of the mu rhythm due to activity of the ‘mirror neurons system’.

We compared mu power between conditions in three groups of participants using rmANOVA (factors: Group, Condition and Hemisphere) and found that it was lowest during the ‘toy-without hand’ condition, intermediate in the ‘toy-without hand’ condition and highest during the ‘social’ condition (Fig.3). All the effects that included the factor Group were not significant (p’s>0.14). As the ‘mirror neuron’ theory would predict highest mu power during attention to the ‘toy without hand’ videos that does not contain human motion, we concluded that in our study the mu power differences were mainly driven by differences in subjects’ motility and/or attentiveness rather then the presence/absence of human motion in the video. Our experimental groups did not differ in either motility, attention or number of segments obtained for each of the experimental conditions. Therefore, we believe that combining across conditions is justified in this study.


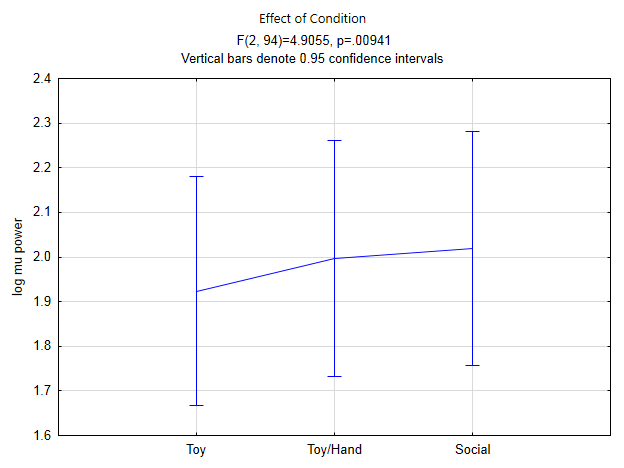


A B C

*Figure S4*. Log mu power during attention to the different types of videos. Only children ho had at least 10 segments of clean EEG data for all three conditions were included into this analysis.

***Group differences in alpha ubPLI (combined conditions): NBS results***

In order to check if weighting of the phase lag index (PLI) by the magnitude of imaginary part of coherence (i.e. WPLI) could principally affect the group differences, the NBS analysis was repeated for the PLI (its unbiased modification, ubPLI (8)). The results obtained with ubPLI (*Fig. S5*) were similar to those obtained with dbWPLI (Fig. 2 A, main article).

*Figure S5.* Networks of increased connections in HR-ASD infants calculated based on ubPLI. The nodes (electrodes) and edges of the hyper-connected networks are loosely modeled on the standard brain image. Similarly to dbWPLI (see main article) the NBS has shown more functional connectivity in infants with ASD (N=10) as compared to both LR (N=26) and HR-no-ASD (N=18) infants.

***Group differences in alpha dbWPLI in social and non-social conditions: NBS results***

For each condition infants who had at least 60 segments of data were included in analysis. For the social condition the EEG data were available for 10 HR-ASD, 17 HR-no-ASD and 23 LR participants. For the non-social condition they were available for 9 HR-ASD, 18 HR-no-ASD and 25 LR infants.

Figure *S6*. Group differences in dbWPLI in social and non-social conditions. The nodes (electrodes) and edges of the hyper-connected networks are loosely modeled on the standard brain image (SPM). Under both conditions NBS showed either significantly elevated connectivity or a tendency for its elevation in infants with ASD as compared with LR infants or HR infants without ASD.

**Lateralization of alpha connectivity measures**

To assess in HR-ASD participants differences in connectivity between the two hemispheres, the average dbWPLI was calculated separately for the electrodes of the left hemisphere, right hemisphere, and between hemispheres. Figure S7 shows similar pattern of group differences for all three types of connections. The results of statistical comparison (Mann-Whitney test) are given in table S6.


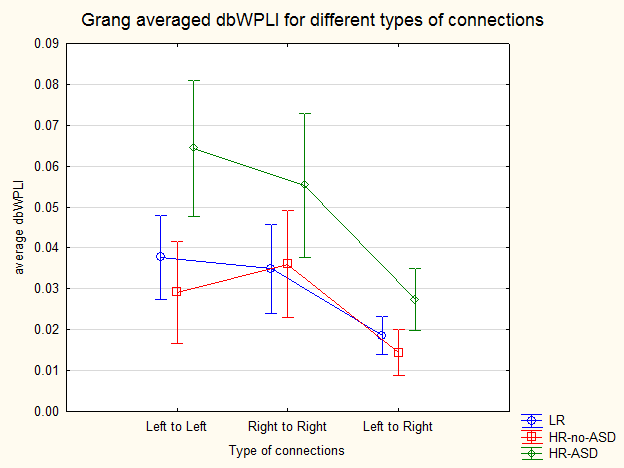


Fig. S7. The average dbWPLI values (95% confidence intervals) for connections within the left hemisphere, right hemisphere, and between hemispheres.

*Table S6*. Group differences in connectivity for different types of connections.

|  | Left to Left | Right to Right | Left to Right |
| --- | --- | --- | --- |
| HR-ASD vs LR | Z=1.89, p=0.06 | Z=1.74, p=0.08 | Z=1.47, p=0.14 |
| HR-ASD vs HR-no-ASD | Z=2.23, p=0.03 | Z=1.65, p=0.09 | Z=2.13, p=0.03 |

When the left- and right-hemispheric connectivity was compared, no hemispheric differences were found for any of the groups. When dbWPLI was averaged according to the regions (Fig. S8) a trend was found in infants with ASD for higher connectivity in the anterior sector of the left hemisphere compared to the symmetrical sector of the right hemisphere (Mann-Whitney, P=0.047, uncorrected for multiple comparisons). No hemispheric differences in dbWPLI were found in the LR or HR-no-ASD groups.

Fig. *S8*. Electrodes used to assess laterality of dbWPLI within anterior, central and posterior sectors. The average dbWPLIs have been calculated for the electrodes of the certain position (e.g. Left anterior) and all other electrodes.

***Effect of gender and developmental level***

*Table S7*. Effect of gender on alpha-range connectivity: means (SD) and probability of male vs. female differences (Mann-Whitney *U*-test; exact two-tailed *P*’s)

|  | GC-dbWPLI* | | | EC-dbWPLI† | | |
| --- | --- | --- | --- | --- | --- | --- |
|  | Male | | Female | Male | Female | |
| LR  (12 males, 14 females) | 0.0304 (0.0189) | | 0.0257 (0.0128) | 0.0357 (0.0273) | 0.0287 (0.0173) | |
|  | *U=69, P* =0.46 | | | *U=73, P* =0.60 | | |
| HR-no-ASD  (3 males, 15 females)‡ | 0.0159 (0.0115) | | 0.0253 (0.0181) | 0.0190 (0.0170) | | 0.0328 (0.0297) |
|  | *U=15, P* =0.43 | | | *U=17, P* =0.57 | | |
| HR-ASD  (7 mails, 3 females)‡ | 0.0381 (0.0247) | 0.0579 (0.0048) | | 0.0905 (0.0574) | | 0.1111 (0.0350) |
|  | *U=8, P* = 0.67 | | | *U=7, P* =0.52 | | |

* GC-dbWPLI - Global Connectivity, i.e. dbWPLI values averaged across all connections;

† EC- dbWPLI values averaged over those connections that were elevated in HR-ASD group compared to both LR and HR-no-ASD groups (see Fig. 2C).

‡ Small group sizes

*Table S8*. Correlations between connectivity measures and MSEL Composite Standard Score.

|  | GA-dbWPLI* | EC-dbWPLI |
| --- | --- | --- |
| LR (N=25) † | *R* = -0.27, *P*=0.2 | *rho* =-0.23, *P* =0.28 |
| HR-no-ASD (N=18) | *rho* =0.01, *P* =0.97 | *rho* =0.03, *P* =0.91 |
| HR-ASD (N=10) | *rho* =-0.03, *P* =0.93 | *rho* =-0.04, *P* =0.92 |

* GC-dbWPLI - Global Connectivity, i.e. dbWPLI values averaged across all connections; EC-dbWPLI values averaged over those connections that were elevated in HR-ASD group compared to both LR and HR-no-ASD groups (see Fig. 2C).

† MSEL was available for all HR infants and for 25 of 26 LR infants. Spearman’s *rho* is given in case for non-Gaussians data distributions (Shapiro-Wilk, *P*<0.05) and Pearson’s *R* is given if the distribution was not different from normal.

***Global alpha connectivity and age***

*Figure S9*. No significant correlations between global alpha-range connectivity and age were found (LR: N=26, Pearson’s *R*=0.02, p=0.94; HR-no-ASD: N=18, Spearman’s *rho*=0.14, p=0.58, HR-ASD: N=10, Spearman’s *rho*=-0.21, p=0.56). Blue – LR; black – HR-no-ASD; red – HR-ASD.

***dbWPLI group differences in short- and long-range connections in the alpha band***

*Figure S10.* Difference between numbers of elevated (Mann-Whitney, P<0.05, uncorrected) connections in HR-ASD group (N=10) and in comparison groups (LR, N=26; HR-no-ASD, N=18). The difference is plotted separately for short range (< 6 cm) and long-range (> 6 cm) connections. For each electrode number of connections that were elevated in the comparison groups comparative to the HR-ASD group is subtracted from the number of connections elevated in the HR-ASD group. Positive values correspond to greater number of elevated connections in HR-ASD infants than in the comparison groups. Negative values correspond to greater number of elevated connections in the comparison groups. Note clustering of over-connected sites over fronto-central regions.

**Supporting References**

1. Mullen EM (1995) *Mullen Scales of Early Learning manual* (American Guidance Service, Circle Pines, MN) AGS Ed pp ix, 85 p.

2. Sparrow SS, Balla DA, Cicchetti DV, & Doll EA (1984) *Vineland adaptive behavior scales : interview edition, survey form manual* (American Guidance Service, Circle Pines, Minn.) pp xiv, 301 p.

3. Lord C, Rutter M, DiLavore PC, & Risi S (1999 ) *Autism diagnostic observation schedule (ADOS)* (Western Psychological Services, Los Angeles) p 140 pages.

4. Lord C, Rutter M, & Lecouteur A (1994) Autism Diagnostic Interview-Revised - a Revised Version of a Diagnostic Interview for Caregivers of Individuals with Possible Pervasive Developmental Disorders. *J Autism Dev Disord* 24(5):659-685.

5. World Health Organization. (1993) *The ICD-10 classification of mental and behavioural disorders : diagnostic criteria for research* (World Health Organization, Geneva) pp xiii, 248 p.

6. Association AP (2013) *Diagnostic and Statistical Manual of Mental Disorders 5th Edition – Text Revision (DSM-5).* (American Psychiatric Association, Washington, DC.).

7. Stam CJ, Nolte G, & Daffertshofer A (2007) Phase lag index: Assessment of functional connectivity from multi channel EEG and MEG with diminished bias from common sources. *Hum Brain Mapp* 28(11):1178-1193.

8. Vinck M, Oostenveld R, van Wingerden M, Battaglia F, & Pennartz CMA (2011) An improved index of phase-synchronization for electrophysiological data in the presence of volume-conduction, noise and sample-size bias. *Neuroimage* 55(4):1548-1565.

9. Vinck M, Battaglia FP, Womelsdorf T, & Pennartz C (2012) Improved measures of phase-coupling between spikes and the Local Field Potential. *J Comput Neurosci* 33(1):53-75.

10. Kuhlman WN (1978) Functional topography of the human mu rhythm. *Electroencephalography and clinical neurophysiology* 44(1):83-93.

11. Rougeul-Buser A, Bouyer JJ, & Buser P (1975) From attentiveness to sleep. A topographical analysis of localized "synchronized" activities on the cortex of normal cat and monkey. *Acta neurobiologiae experimentalis* 35(5-6):805-819.

12. Oberman LM & Ramachandran VS (2007) The simulating social mind: the role of the mirror neuron system and simulation in the social and communicative deficits of autism spectrum disorders. *Psychological bulletin* 133(2):310-327.
